# Supplementary material for: The Prognostic Value of the Systemic Immune-Inflammation Index (SII) and Red Cell Distribution Width (RDW) in Patients with Cervical Cancer Treated Using Radiotherapy
Source: Cancers (Basel). 2024 Apr 18;16(8):1542. doi: 10.3390/cancers16081542 (PMC11049631; doi:10.3390/cancers16081542)
Supplement: Supplementary file 1 [file cancers-16-01542-s001.zip › cancers-2924666-supplementary.pdf]

Supplementary file

Table S1. The irradiation schemes of external beam radiotherapy (EBRT) used in the treatment of 249 patients receiving definitive chemoradiation therapy for cervical cancer.

**EBRT**	
**Dc/df + boost EBRT [Gy]**	**N**
30/3 and 21/3*	1
44/2 + 10/2	1
45/1.8	13
45/1.8 + 5.4/1.8	1
45/1.8 + 5.4/1.8 + 6/2	1
45/1.8 + 6/2	1
45/1.8 + 9/1.8	7
45/1.8 + 9/1.8 + 6/2	2
45/1.8 + 14.4/1.8	3
45/1.8 + 19.8/1.8	1
46/2 + 18/2	1
50.4/1.8	113
50.4/1.8 + 3.6/1.8	27
50.4/1.8 + 5.4/1.8	13
50.4/1.8 + 6/2	1
50.4/1,8 + 9/1.8	9
50.4/1.8 + 10/2	2
50.4/1.8 + 10.8/1.8	1
50/2	2
54/1.8	36
54/1.8 + 3.6/1.8	6
54/1.8 + 6/2	4
54/1.8 + 14.4/1.8	2

54.6/1.95	1
	**249 (100%)**

Abbreviations: EBRT – external beam radiotherapy; Dc – total dose; df – fractionation dose; N – number of patients; *scheme of definitive EBRT in two phases.

Table S2. The irradiation schemes of brachytherapy (BT) used in the treatment of 246 patients receiving definitive chemoradiation therapy for cervical cancer.

**BT**		
**Dc/df [Gy]** (I phase + II phase)	**Prescription point** (I phase + II phase)	**N**
5/5*	Point A	2
15/7.5	Basal points	1
20/5	Point A	46
20/10	1cm from probe's axis	1
25/5	Point A	169
30/5	Point A	2
40/5	1cm from plate's surface	1
40/8	1cm from plate's surface	5
40/10	1cm from probe's axis	9
50/10	1cm from probe's axis	2
20/5 + 14/7	Point A + 0,5cm from needle's axis in all directions (interstitial-boost)	1
25/5 + 14/7	Point A + 0,5cm from needle's axis in all directions (interstitial-boost)	1
20/10 + 15/5	1cm from probe's axis + Point A	1
25/5 + 30/10	0,8cm from applicator's surface (multichannel cylinder) + 1cm from probe's axis	1
32/8 + 30/10	1cm from plate's surface + 1cm from probe's axis	2
40/8 + 33/11	1cm from plate's surface + 1cm from probe's axis and ring's surface	1
24/8 + 40/10	1cm from plate's surface + 1cm from probe's axis and ring's surface	1
		**246 (98.8%)**
Prescription points according to applicator type:<br><br>Point A – tandem applicator		

Basal points – interstitial implant
1cm from probe's axis – single-channel intrauterine applicator
1cm from plate's surface – individual applicator

Abbreviations: BT – brachytherapy; Dc – total dose; df – fractionation dose; N – number of patients; *treatment interrupted due to the bad condition or patient's decision.
